# Supplementary material for: Deteriorated sleep quality and associate factors in patients with type 2 diabetes mellitus complicated with diabetic peripheral neuropathy
Source: PeerJ. 2024 Jan 22;12:e16789. doi: 10.7717/peerj.16789 (PMC10809979; doi:10.7717/peerj.16789)
Supplement: Supplemental Information 1 [file peerj-12-16789-s001.pdf]

编号________　　　　　　　　床号________　　　　　　调查日期________

II 型糖尿病相关情况调查表

亲爱的病友朋友：

您好！下面这些问题只围绕您的生活、睡眠及疾病等情况，如您同意请按照自身实际情况填写下面的资料，为更好地治疗和康复提供参考。本次调查不记名，我们承诺对您的资料严格保密，感谢您的配合！

一、一般情况

1.0 性别： ①男　②女

1.1 年龄：_____岁　　　1.2 身高：_____cm　　　1.3 体重：_____Kg

1.4 您的常住地：①城市　　②乡镇　　③农村

1.5 您的婚姻状况：①已婚　②未婚　③其他（离异、丧偶）

1.6 您的职业：①农民　②工人 ③企事业单位工作者　④做生意　⑤退休　⑥其他

1.7 您的文化程度：①小学及以下　②初中　③高中及中专　④大专及以上

1.8 您的家庭月收入：①2000 以下　②2000-5000　③5001-8000　④8000 以上

二、影响因素

2.0 您之前做过手术吗？ ①否　②是

2.1 您是否患有高血压？ ①否　②是

2.2 您是否坚持服药进行治疗？ ①是　②否

2.3 您目前感觉身体有疼痛吗？ ①没有　②有

2.4 病程长短：①<1 年　②1-5 年　③>5 年

2.5 您是否吸烟：①是　②否　③已戒烟

2.6 您是否饮酒：①是　②否　③已戒酒

2.7 您目前的饮食状况如何？ ①很好　②一般　③很差

2.8 您多久体检一次？ ①半年　②一年　③两年　④从不主动体检

2.9 为了治病，您有经济负担吗？ ①完全无　②基本无 ③有一些　④很重

2.10 您一日三餐规律吗？ ①很规律　②比较规律　③不怎么规律　④很不规律

2.11 您感觉工作或生活压力很大吗？①从未　②很少　③有时　④经常　⑤总是

2.12 您平时运动吗？ ①5 次以上/周　②3-4 次/周　③1-2 次/周　④几乎不运动

2.13 该疾病给您带来了很大的心理负担吗？①没有 ②稍有 ③中度 ④较大 ⑤严重

2.14 空腹血糖_____　　2.15 餐后两小时血糖_____　　2.16 糖化血红蛋白_____

2.17 空腹 C 肽_____

三、睡眠情况（请根据您最近一个月的情况，回答下列问题）

1、近一个月，晚上睡觉通常是几点：_____

2、每晚入睡通常需要_____分钟

3、通常早上_____点起床

4、每夜通常实际睡眠_____小时（不等于卧床时间）

对下列问题请选择数字为每个问题最合适的答案

5、近一月，因下列情况影响睡眠而烦恼：	①没有（月）	②平均少于 1 次（周）	③平均 1-2 次（周）	④平均3次及以上（周）
a.入睡困难（30 分钟内不能入睡）				
b.夜间易醒或早醒				
c.夜间去厕所				
d.呼吸不畅				
e.咳嗽或鼾声高				
f.感觉冷				
g.感觉热				
h.做噩梦				
i. 疼痛不适				
j.其他影响睡眠的事情				
如有，请说明				
6、您用催眠药的情况				
7、您常感觉到困倦吗？				
8、您做事情精力不足吗？				

9、近一月，总的来说，您认为自己的睡眠：①很好　②较好　③较差　④很差
